# Supplementary material for: Exploring the barriers and facilitators to accessing and utilising mental health services in regional, rural, and remote Australia: A scoping review protocol
Source: PLoS One. 2022 Dec 9;17(12):e0278606. doi: 10.1371/journal.pone.0278606 (PMC9733872; doi:10.1371/journal.pone.0278606)
Supplement: S1 File — (DOCX) [file pone.0278606.s002.docx]

**S2. Grey literature information sources.**

| **Source** | **Website** |
| --- | --- |
| *Australian Commonwealth and State/Territory Websites* | |
| Australian Government Department of Health | <https://www.health.gov.au/> |
| Victoria State Government, Department of Health and Human Services | <https://www.dhhs.vic.gov.au/> |
| New South Wales State Government, Department of Health | <https://www.health.nsw.gov.au/> |
| Government of South Australia, South Australia Health | <https://www.sahealth.sa.gov.au/wps/wcm/connect/public+content/sa+health+internet/home/home> |
| Northern Territory Government, NT Health | <https://health.nt.gov.au/> |
| Government of Western Australia, Department of Health | <https://ww2.health.wa.gov.au/> |
| Tasmanian Government, Department of Health | <https://www.health.tas.gov.au/> |
| Australian Capital Territory, Health | <https://www.health.act.gov.au/> |
| *Primary Health Networks* |  |
| Central and Eastern Sydney | <https://www.cesphn.org.au/> |
| Northern Sydney | <https://sydneynorthhealthnetwork.org.au/> |
| Western Sydney | <https://wentwest.com.au/> |
| Nepean Blue Mountains | <https://www.nbmphn.com.au/> |
| South Western Sydney | <https://www.swsphn.com.au/> |
| South Eastern NSW | <https://www.coordinare.org.au/> |
| Western NSW | <https://www.wnswphn.org.au/> |
| Hunter New England and Central Coast | <https://thephn.com.au/> |
| North Coast | <https://hnc.org.au/> |
| Murrumbidgee | <https://mphn.org.au/> |
| North Western Melbourne | <https://nwmphn.org.au/> |
| Eastern Melbourne | <https://www.emphn.org.au/> |
| South Eastern Melbourne | <https://www.semphn.org.au/> |
| Gippsland | <https://www.gphn.org.au/> |
| Murray | <https://www.murrayphn.org.au/> |
| Western Victoria | <https://westvicphn.com.au/> |
| Brisbane North | <https://brisbanenorthphn.org.au/> |
| Brisbane South | <https://bsphn.org.au/> |
| Gold Coast | <https://gcphn.org.au/> |
| Darling Downs and West Moreton | <https://www.ddwmphn.com.au/> |
| Western Queensland | <https://www.wqphn.com.au/> |
| Central Queensland, Wide Bay, Sunshine Coast | <https://www.ourphn.org.au/> |
| Northern Queensland | <https://www.nqphn.com.au/> |
| Adelaide | <https://adelaidephn.com.au/> |
| Country South Australia | <https://www.countrysaphn.com.au/> |
| Western Australia | <https://www.wapha.org.au/> |
| Tasmania | <https://www.primaryhealthtas.com.au/> |
| Northern Territory | <https://www.ntphn.org.au/> |
| Australian Capital Territory | <https://www.chnact.org.au/> |
| *Rural and/or remote associations* |  |
| Services for Australian Rural and Remote Allied Health | <https://sarrah.org.au/> |
| Rural and remote health | <https://www.rrmh.com.au/> |
| National Rural Health Alliance | https://www.ruralhealth.org.au/ |
| Australian Rural Health Education Network | <https://www.arhen.org.au/> |
| *Other* |  |
| Australian Institute of Health and Welfare | <https://www.aihw.gov.au/> |
| Australian Health Services Research Institute | <https://ahsri.uow.edu.au/> |
| informIT | via Deakin University |
| Australian Indigenous HealthInfoNet | <https://healthinfonet.ecu.edu.au/> |
| Suicide Prevention Australia | [https://www.suicidepreventionaust.org/#](https://www.suicidepreventionaust.org/) |
| University Departments of Rural Health | <https://arhen.org.au/> |
| Global Health Data Exchange | <http://ghdx.healthdata.org/> |
| Google | <https://www.google.com/> |
